# Perception of *Enterococcus faecalis* without infection induces *fmo-2* in *C. elegans*

**DOI:** 10.17912/micropub.biology.001422

**Published:** 2025-01-10

**Authors:** Megan L Schaller, Madeline M Sykes, Sarah A Easow, Faith R Carranza, Angela M Tuckowski, Yatrik M Shah, Scott F Leiser

**Affiliations:** 1 Molecular and Integrative Physiology Department, University of Michigan–Ann Arbor, Ann Arbor, Michigan, United States; 2 Department of Molecular and Cellular Pathology, University of Michigan–Ann Arbor, Ann Arbor, Michigan, United States; 3 Cellular and Molecular Biology Program, University of Michigan–Ann Arbor, Ann Arbor, Michigan, United States

## Abstract

*C. elegans* pathogenic susceptibility is influenced by the worm’s detection of its environment and its capacity to resist and resolve damage following infection. Here, we use a model where worms can sense, but not ingest, the pathogen *Enterococcus faecalis* (EF) *.* We identify that perception of EF without infection induces the stress-response gene *fmo-2. We* further identify that neural and intestinal signaling genes are necessary for *fmo-2* induction without active infection. Finally, we show that *fmo-2* overexpression is sufficient to extend lifespan with EF exposure, while *fmo-2* KO is not detrimental, suggesting that additional *fmo-2* expression benefits worms in this condition.

**Figure 1. fmo-2 induction with EF Smell involves neural and intestinal signaling pathways:**
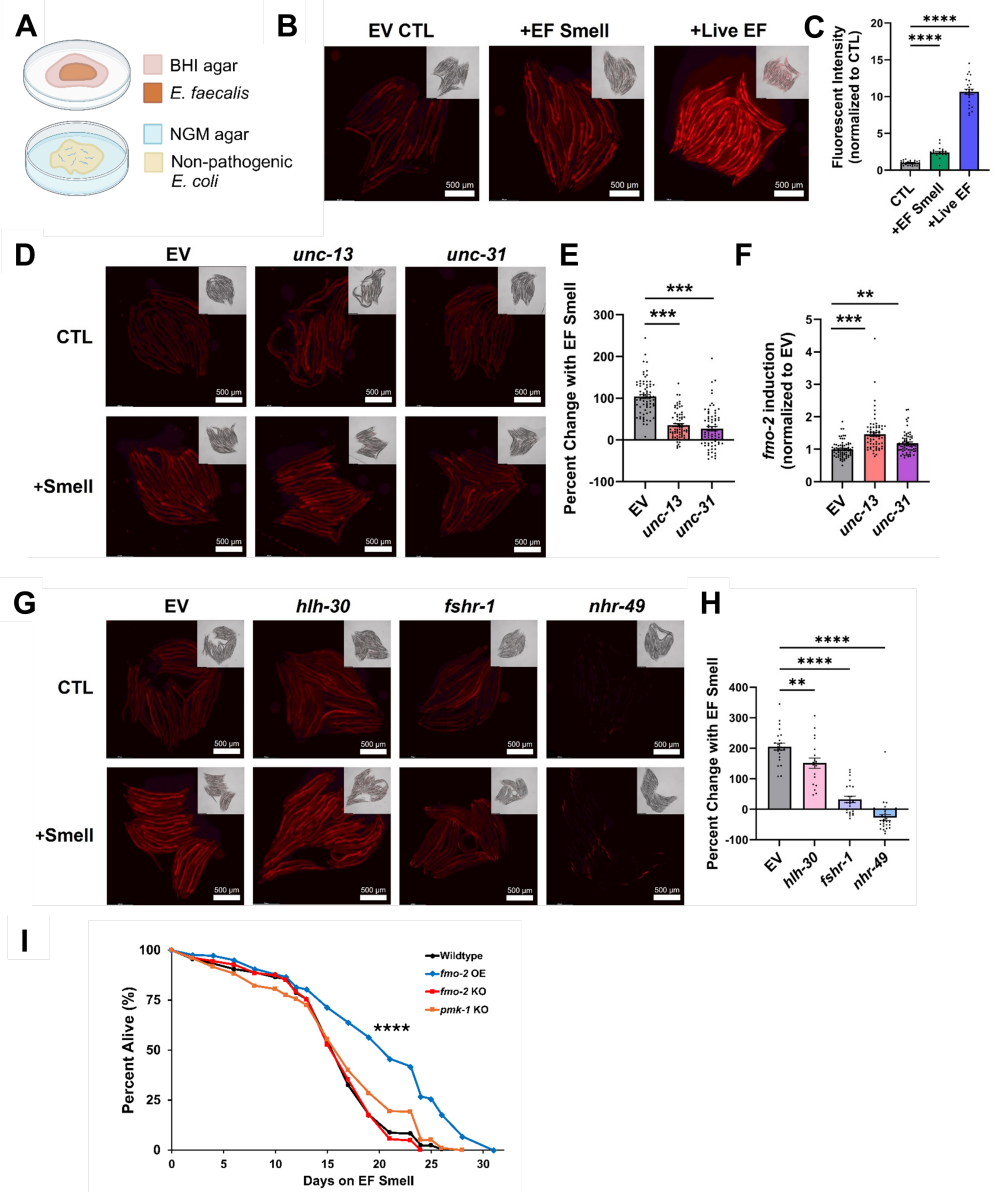
( **A** ) Schematic depicting the EF Smell condition: worms reside on a lawn of wildtype OP50 *E. coli* on NGM agar and on the lid of the plate live *E. faecalis* is seeded onto BHI agar. ( **B** ) Fluorescent images of *fmo-2p::mcherry* worms on day 2 of adulthood showing *fmo-2* induction after 24 hours at 25°C on Empty Vector (EV) CTL, +EF Smell, or +Live EF conditions (23-25 worms/condition). ( **C** ) Fluorescent intensity quantified for each worm using ImageJ and normalized to average of the CTL group. ( **D** ) Images of *MAH677::fmo-2p::mcherry* worms fed respective RNAi bacteria for two generations prior to exposure to EF Smell (or CTL) for 24 hours. ( **E** ) Percent change of fluorescent intensity with EF Smell calculated for each type of RNAi. ( **F** ) *fmo-2* induction in non-challenged worms, measured by fluorescent intensity and normalized to EV condition. (Includes data points from 3 independent replicates, with 22-27 worms/condition each replicate). ( **G** ) Images of *fmo-2p::mcherry* worms fed RNAi bacteria for one generation prior to CTL or EF Smell exposure for 24 hours. Statistical significance for **C** , **E, F** , and **H** was calculated using a 1-way ANOVA with Brown-Forsythe test and Dunnett’s T3 correction for multiple comparisons where appropriate. ( **I** ) Lifespan curve of Wildtype, *fmo-2* OE, *fmo-2* KO, and *pmk-1* KO worms exposed to EF Smell at 25°C for duration of lifespan. Graph displays a combination of data from two individual replicates (60-80 worms per technical replicate, 2 technical replicates per biological replicate). Statistical significance was calculated using log-rank analysis and relevant significant differences are displayed on graph. * = p < 0.05, ** = p < 0.01, *** = p < 0.001, **** = p < 0.0001.

## Description

The ability to detect and respond to pathogens in an organism’s environment is critical for survival. With a fully mapped nervous system (Cook et al., 2019) , the nematode *Caenorhabditis elegans* provides a valuable model for investigating sensory detection of pathogens and host defense. Previous work supports that many of the neural and immune signaling pathways of worms are conserved across taxa (Hoffmann et al., 1999) . Additionally, the *C. elegans* diet of live bacteria provides the opportunity to dissect the nuanced molecular mechanisms involved in pathogen response.

The *C. elegans* immune response is multifaceted and tailored to the physical and chemical attributes of each pathogen as well as the mode of infection (Irazoqui et al., 2010a, Martineau et al., 2021) . Sensory detection of virulence factors or toxins produced by pathogens and recognition of the physical or structural components of pathogens, such as cell wall composition, are utilized to detect pathogenic stress prior to ingestion of the bacteria (Akira et al., 2006) . Following ingestion of a pathogen, *C. elegans* can sense perturbations to key biological processes such as protein translation and mitochondrial homeostasis, a phenomenon referred to as surveillance immunity (Pukkila-Worley, 2016) . Worms also detect pathogen-induced tissue damage resulting from infection and immune activation under these circumstances triggers a signaling cascade focused on resolving and clearing the infection (Irazoqui et al., 2010a). This post-ingestion type of immune response is thought to be both distinct from and additive with the preventative/proactive transcriptional profile induced by detecting a pathogen without consumption (Engelmann and Pujol, 2010) . The capacity to recognize a wide range of bacterial, viral, and fungal threats is critical for *C. elegans* survival, although the mechanisms and intricacies of pathogen recognition are still being defined.

Bacterial invasion of intestinal cells and subsequent colonization is the primary method used by intestine-targeting pathogens in *C. elegans* , making intestinal health/defense a key factor in host resistance to pathogens (Garsin et al., 2001) . The intestinal enzyme flavin-containing monooxygenase-2 ( *fmo-2* ) is implicated in environmental stress resistance and longevity in *C. elegans* and plays a role in xenobiotic and endogenous metabolism (Leiser et al., 2015; Huang et al., 2021) . *fmo-2* was initially implicated in *C. elegans* immunity from a transcriptional profiling screen as being greatly induced with *S. aureus* infection (Irazoqui et al., 2010b). Further investigation revealed that *fmo-2* was induced by both live and dead *S. aureus,* as well as with the pathogen *Enterococcus faecalis* (EF) (Irazoqui et al., 2010b). *E. faecalis* is a Gram-positive cocci bacterium that colonizes and proliferates in the intestine of worms when ingested, shortening lifespan (Garsin et al., 2001) . Additional work found that knockdown of *fmo-2* by RNA-mediated interference (RNAi) increases susceptibility of worms to EF (Dasgupta et al., 2020) . These findings highlight an important role for *fmo-2* in pathogenic resistance, with limited evidence as to its mechanism in innate immunity.

Here, we utilize a model where worms are exposed to live *E. faecalis* on the lid of an agar plate but are unable to consume the pathogen (referred to as EF Smell). We hypothesized that EF smell could be sufficient to turn on an immune response and induce *fmo-2,* and if it did, we could use this induction to identify key genes necessary for the effect, in comparison to those involved in active infection. A diagram of our approach is depicted in **Figure 1A** . Using a transcriptional fluorescent *fmo-2* reporter worm strain ( *fmo-2p::mcherry* ) (Miller et al., 2022) , we find that *fmo-2* is significantly induced after 24 hours of exposure to both EF Smell and live *E. faecalis* , compared to unchallenged control worms (CTL) ( **Figure 1B -C** ). The induction of *fmo-2* under EF smell is notably less than when infected with EF, consistent with multiple sensory and damage pathways that induce *fmo-2* under infection being less activated by EF smell.

Because the intestine is the primary organ responsible for *C. elegans* immunity (Wani et al., 2020) , but the nervous system mediates most environmental perception, bidirectional communication between the nervous system and the intestine is required to produce an appropriate immune response (Liu and Sun, 2017) . Select neural pathways are involved in neural-to-intestine signaling in response to a variety of pathogens (Wani et al., 2020) . The lack of innervation into the intestinal epithelium in both *C. elegans* and mammals suggests that neural signals are communicated to the intestine through long range signals including neurotransmitters, neuropeptides, or chemical signals (Kaelberer et al., 2018) . To test the necessity of genes associated with synaptic vesicle fusion and neurotransmitter release ( *unc-13* ) (Richmond et al., 1999) or neuropeptide secretion ( *unc-31* ) (Speese et al., 2007) , we used RNAi to knock down these genes in a *fmo-2* transcriptional reporter strain with RNAi machinery only in the nervous system *MAH677 (sid-1(qt9);sqIs71[rgef-1p::GFP + rgef-1p::sid-1]);fmo-2p::mcherry* ). We find that *unc-13* and *unc-31* RNAi each significantly blunt the induction of *fmo-2* by 24H of exposure to EF Smell ( **Figure 1D -F** ). This is consistent with *fmo-2* being induced by multiple types of neural signaling during EF Smell exposure. This capability aligns with previous work supporting that *fmo-2* is induced with a variety of environmental stressors (hypoxia, dietary restriction, pathogen exposure, etc.), frequently cell non-autonomously (Irazoqui et al., 2008; Leiser et al., 2015; Huang et al., 2021) .

Regarding intestinal signaling, there are a limited number of transcription factors identified as regulators of *fmo-2* during pathogen response. These include 1) *hlh-30* / *TFEB* , induced under nutritional deprivation and infection and has been implicated in lipid utilization and autophagy (O’Rourke and Ruvkun, 2013; Visvikis et al., 2014), 2) *fshr-1* (mammalian follicle stimulating hormone receptor homolog) which is necessary for *fmo-2* induction with consumption of both *E. faecalis* and the non-virulent strain *Enterococcus faecium* , suggesting *fshr-1* regulation of *fmo-2* occurs independently of infection-induced damage (Yuen and Ausubel, 2018) and 3) *nhr-49/* PPARα (Van Gilst et al., 2005) *,* which is involved in the utilization of fat and fatty acid saturation during pathogen exposure and whose requirement for *fmo-2* induction is reported during dietary restriction, oxidative stress, and active *S. aureus* and *E. faecalis* infections *(* Goh et al., 2018; Wani et al., 2020; Dasgupta et al., 2020). Consistent with the findings of Wani et al. using *S. aureus* infection, we find that *fmo-2* induction by EF smell ( *fmo-2p::mcherry* strain *)* is partially suppressed by *hlh-30* RNAi ( **Figure 1G -H** ), suggesting partial regulation of *fmo-2* by *hlh-30* is not specific to just *S. aureus* nor just with active infection. Our results with *fshr-1* knockdown show a near complete suppression of *fmo-2* induction with EF Smell ( **Figure 1G -H** ), suggesting that *fshr-1* regulates *fmo-2* even in the absence of *Enterococci* consumption. This is contrary to Gupta et al. who concluded *fmo-2* induction with EF was not regulated by *fshr-1* . Finally, we find that *nhr-49* knockdown completely blunts *fmo-2* induction with exposure to EF Smell ( **Figure 1E** ). Together, these results indicate that the transcriptional regulation of intestinal *fmo-2* with sensory exposure to EF without ingestion follows a similar pattern to the regulation of *fmo-2* during active infection. This highlights the capacity of *fmo-2* to respond to EF independently of infection-associated damage.

Lastly, we tested how EF smell affects the lifespan of *fmo-2* knockout and overexpressing animals. Using *pmk-1 (KU25)* mutants (Inoue et al., 2005) as a control to show there is no infection (these animals are short-lived when infected), we fed wildtype, *fmo-2* knockout (KO) ( *ok2147)* (Eom et al., 2013) , and *fmo-2* overexpressing (OE) ( *KAE9* ) (Leiser et al., 2015) worms *E.* *coli* OP50 bacteria while being exposed to live EF on the lid of agar plates continuously starting at day 1 of adulthood. We find that *fmo-2* OE worms live significantly longer than other strains under EF Smell conditions ( **Figure 1I** ), whereas *fmo-2* KO animals are normal-lived (p=0.492), contrary to their lifespan when infected by EF. The result that *fmo-2* is not necessary but **is** sufficient for extended lifespan on EF Smell conditions suggests that the response to EF smell is limited enough that lifespan is not greatly affected. This result warrants future studies as to the outcomes of differing cellular responses between sensory perception of *E. faecalis* and active infection.

## Methods

**C. elegans Strains and Maintenance**

Worms were grown and maintained on either solid nematode growth media (NGM), NGM with 1 mM β-D-isothiogalactopyranoside (IPTG) (Fisher Scientific) and 25 μg/ml carbenicillin (Fisher Scientific) (all RNAi experiments), or NGM with 150 mM fluorodeoxyuridine (FUdR) added (EF Smell Lifespans). All worms used for experiments were age synchronized by timed egg lay and grown to day 1 of adulthood prior to experimental intervention. Worms were incubated at 20° Celsius (C) except during EF and EF Smell exposure, when they were incubated at 25° C. RNAi experiments were performed with LZR1 ( *fmo-2p::mcherry* ) or *MAH677 (sid-1(qt9);sqIs71[rgef-1p::GFP + rgef-1p::sid-1]);fmo2p::mcherry* (neural-specific RNAi machinery) worms. EF Smell lifespans were performed with Wildtype N2, KAE9 ( *fmo-2* OE), VC1668 ( *fmo-2* KO), and KU25 ( *pmk-1* KO) worm strains. All experimental plates contained a fence of palmitic acid to prevent worm fleeing.

**Live and Smell *E. faecalis* Exposure and Imaging**

A flask of 1x Brain Heart Infusion Broth (BHI, BD Difco) with 100 μg/mL Rifampicin (Thermo Scientific Chemical) was inoculated with the pathogen *E faecalis* OG1RF (ATCC) and cultured for 18 hours shaking at 37° C. 100 μL of the resulting 1x *E. faecalis* culture was seeded either onto 35 mm BHI agar (BD Difco) + Rifampicin plates (Live EF condition) or 1 mL of BHI agar + Rifampicin on the lid of 60 mm plates (EF Smell conditions), before plates were incubated at 37° C for 18 hours. After incubation, all EF and EF Smell plates cooled to room temperature prior to worms being added. Worms were transferred to fresh EF or EF smell plates beginning at day 1 of adulthood and were incubated at 25° C for 24 hours (RNAi experiments) or for the duration of lifespan (EF Smell lifespans). Controls for EF and EF Smell experiments were kept in a separate incubator (to avoid untargeted EF exposure), also at 25° C and starting at day 1 of adulthood.

**RNAi Imaging Experiments**

*E. coli* HT115 bacterial strains expressing double stranded RNA for genes used in knockdown imaging experiments were obtained from Vidal and Ahringer *C. elegans* RNAi libraries. Individual RNAi plates (including Empty Vector as Control) were seeded with 2x concentrated RNAi bacteria. *MAH677 (sid-1(qt9);sqIs71[rgef-1p::GFP + rgef-1p::sid-1]);fmo2p::mcherry* worms were age synchronized by timed egg lay onto RNAi plates and grown for two generations prior to EF Smell (and CTL) exposure on day 1 of adulthood for 24 hours. *fmo-2p::mcherry* worms were grown on respective RNAi plates for one generation prior to EF Smell exposure. Since the EF Smell condition only contained BHI agar and live EF on the lid of the agar plate, the base of agar plates contained NGM+IPTG and carbenicillin and were seeded with 2x RNAi bacteria. To image, worms were transferred from plates to a 3% agarose pad and immobilized with 1M sodium azide before a glass coverslip was added and images were taken using a Leica fluorescent microscope. The fluorescent intensity of each worm was quantified using Image J software.

**EF Smell Lifespans**

Wildtype, *fmo-2* OE, *fmo-2* KO, and *pmk-1* KO worms were age synchronized by timed egg lay onto NGM agar plates seeded with 1x *E. coli* OP50. On day 1 of adulthood, worms were transferred to NGM + FUDR plates that were seeded with 5x *E. coli* OP50 and had live EF on the lid (EF Smell condition). Plates were incubated at 25° C for the duration of the lifespan and the survival of worms was scored every other day. Each strain was run in duplicate and plate lids with EF were exchanged for fresh EF lids every 3-4 days.

**Statistical Analyses**

All imaging data is displayed as mean +/- SEM. The sample size of worms in each experiment are in the figure legend. Data shown is representative of at least 3 individual replicates. All plots were graphed and statistical tests were calculated using GraphPad Prism Version 10.2.2 except for the lifespan curve, which was plotted in Excel and analyzed using R Studio. Statistical significance for imaging data was calculated by 1-way ANOVA with Brown-Forsythe test and Dunnett’s T3 correction for multiple comparisons where appropriate. Lifespan curve results are a culmination of 2 individual replicates and significant differences were determined by log-rank test. Statistical significance was determined for p-values less than 0.05, and are reported as * when p < 0.05, ** when p < 0.01, *** when p < 0.001, and **** when p < 0.0001. **Figure 1A** image was created using BioRender. All authors had access to study data and have reviewed, edited, and approved the final manuscript.
